# The Hemobag: the modern ultrafiltration system for patients undergoing cardiopulmonary by pass

**DOI:** 10.1186/1749-8090-7-55

**Published:** 2012-06-14

**Authors:** Andrea Colli, Sara Balduzzi, Xavier Ruyra

**Affiliations:** 1 Department of Cardiac Surgery, Hospital Universitari Germans Trias i Pujol, Badalona, Spain; 2 Department of Cardiac, Thoracic and Vascular Sciences, University of Padova, Via Giustiniani 2, Padova, 35100, Italy; 3 Department of Oncology, Hematology and Respiratory Diseases, University of Modena and Reggio Emilia, Modena, Italy

**Keywords:** Ultrafiltration, MUF, Cardiopulmonary bypass, Autologous blood conservation, Cardiac surgery

## Abstract

**Background:**

The return of extracorporeal circuit blood at the termination of cardiopulmonary bypass (CPB) is an important feature of blood conservation during cardiac surgery procedures globally. We report our initial clinical evaluation of the Hemobag system a blood-salvaging device designed for whole blood recovery of residual post-CPB volume**.**

**Methods:**

Residual whole blood is hemoconcetrated through the multipass “recovery loop” circuit separate from the CPB and collected in the Hemobag System. This allows the surgeons to continue with surgery, decannulate, and administer protamine simultaneously while the Hemobag is in use and the CPB circuit remains safely primed. We have compared 25 patients receiving the Hemobag to a control group of 25 patients treated with the cell washer that represented our previous standard of care method of circuit blood-salvaging technique.

**Results:**

The Hemobag system provided significantly higher hemoglobin, hematocrit, fibrinogen, albumin, and total protein levels in the final product reducing the amount of wasted autologous blood cells. There were no device-related complications. There were no significant differences in terms of blood utilization, chest tube drainage and clinical outcomes over the entire postoperative period among groups.

**Conclusions:**

These results suggest that the Hemobag system is a safe and efficient method to multipass hemoconcentrate the residual diluted blood of the CPB circuit. The Hemobag has demonstrated its ability to maximize the composition of the residual CPB volume to achieve the best possible post-CPB hemoglobin, plasma protein and coagulation factors profile for the patient respect to CW.

## Background

Cardiac surgery is one of the leading consumers of blood products. Intraoperative and postoperative blood losses are predictable contributors to this problem. A less commonly recognized factor is the effect of hemodilution of the patient by the pump prime of cardiopulmonary bypass (CPB) circuit.

Blood conservation and fluid management are now the most important issues surrounding cardiac surgery today. Recent data independently link allogeneic blood use to increase morbidity and mortality after CPB [1,2].

Following termination of bypass, the CPB circuit contains a significant volume of diluted blood. The CPB is capable of containing a significant amount of diluted residual autologous whole blood. Commonly, this residual blood is discarded or only partially salvaged for a number of reasons including 1) the blood is excessively dilute, 2) it contains a significant amount of activated mediators, and 3) the notion that the platelets are dysfunctional and subsequently impair overall coagulation status [2,3].

Published data have shown that most of the ill effects of CPB on platelets and other coagulation factors are temporary and reversible within hours post-operatively [4]. The hemodilution encountered during cardiac surgery can be corrected through hemoconcentration [5].

Various methods have been used to salvage this blood, including centrifugation/washing, direct transfusion and ultrafiltration both on-CPB and post-CPB. The centrifugation/washing technique produces a reinfusion product that is free of plasma proteins, coagulation factors, and platelets [5].

The direct infusion into the patient cause hemodilution and volume overload, contributing to organ edema and organ dysfunction and requiring vasodilatation and diuretic therapy to control these negative effects that can last 4–8 hours postoperatively. Subsequently, this can create further hemodynamic instability and electrolyte imbalance [6].

Alternatively ultrafiltration has the advantage of removing excess water volume, which has been shown to improve, hematocrit, arterial oxygen content concentration of coagulation factors, and decreasing tissue edema and organ dysfunction [7]. Low molecular weight components, which may include cytokines and toxins, are also removed owing to the membrane pore size, thus potentially decreasing perioperative inflammation [8].

Ultrafiltration during CPB and post-CPB has evolved into different techniques along decades such as zero-balance ultrafiltration and modified ultrafiltration. Established ultrafiltration techniques are time consuming, requiring the cannulae to remain in the patient during the entire process, and thus delaying the reversal of heparinization.

The Hemobag system (Global Blood resources LLC, Somers, CT) has been developed to offset the difficulties associated with standard ultrafiltration techniques.

We report our initial evaluation and clinical experience with the Hemobag ultrafiltration system.

## Methods

### Study population

Consecutive patients scheduled for elective cardiac surgery with the use of CPB at the Hospital Universitari Germans Trias i Pujol, Badalona, Spain, were selected. Patients were divided into the Hemobag group (Group H) and in the Cell washer group (Group CW) according to the Surgeon preference. We included patients who were undergoing CABG surgery or cardiac valve replacement or repair, alone or in combination. Exclusion criteria were: patients over 80 years old, redo or emergency surgery, endocarditis or pericardic disease.

We have compared 25 patients receiving the Hemobag (Group H) to a matched control group of 25 patients treated with the CW that represented at that time our standard of care method to process the residual CPB circuit blood.

At the end of surgery all remaining blood inside the CPB circuits was recovered and concentrated by the CW (Electa, Sorin group, Saluggia, Italy) if patient was in Group CW. In case patient was in Group H, the remaining blood was recovered and ultrafiltrated using the Hemobag System.

In the Group H all blood in the surgical field was aspirated only using the cardiotomy suction and in Group CW was aspirated using both the CW and the cardiotomy suction. All blood was recovered and concentrated/ultrafiltrated before starting protamine. After reinfusion of the recovered blood an extra dose of protamine (20% of the standard dose) was administered in all patients. The cardiopulmonary bypass protocol and equipment used were identical for all patients enrolled in the study. Local review board approved the study. Written informed consent was obtained from the patients. The present clinical research was carried out in compliance with the Helsinki Declaration.

### Technology

The Hemobag system received is commercially available in Europe, United States of America and Canada. The Hemobag recovers autologous whole blood at the end of CPB with all the cells and proteins still intact. The Hemobag system concentrates the residual CPB volume with multipass hemoconcentration to a target volume or target hematocrit decided by the surgical team. The CPB is always maintained safely primed and ready to immediately go back on bypass if there is an emergency. The Hemobag circuit is separate from the CPB circuit. This allows the surgeons to continue with surgery, decannulate, and administer protamine simultaneously while the Hemobag is in use.

**Figure 1:**
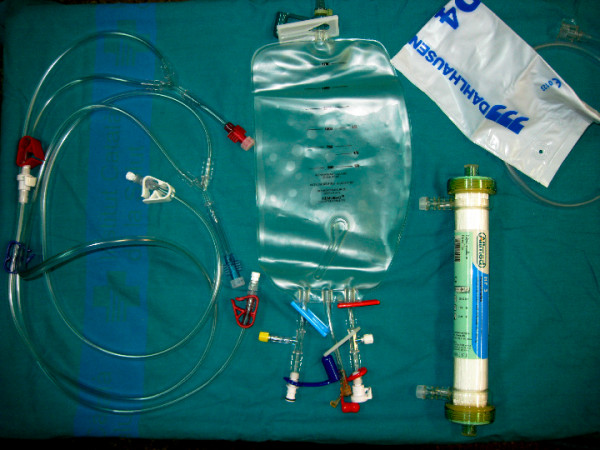
Hemobag kit components.

The Hemobag system kit is composed of the Hemobag, the TS3 tubing set (Global Blood Resources), and hemoconcentrator (Minntech HPH 1400; Minneapolis, MN) Figure 1.

**Figure 2:**
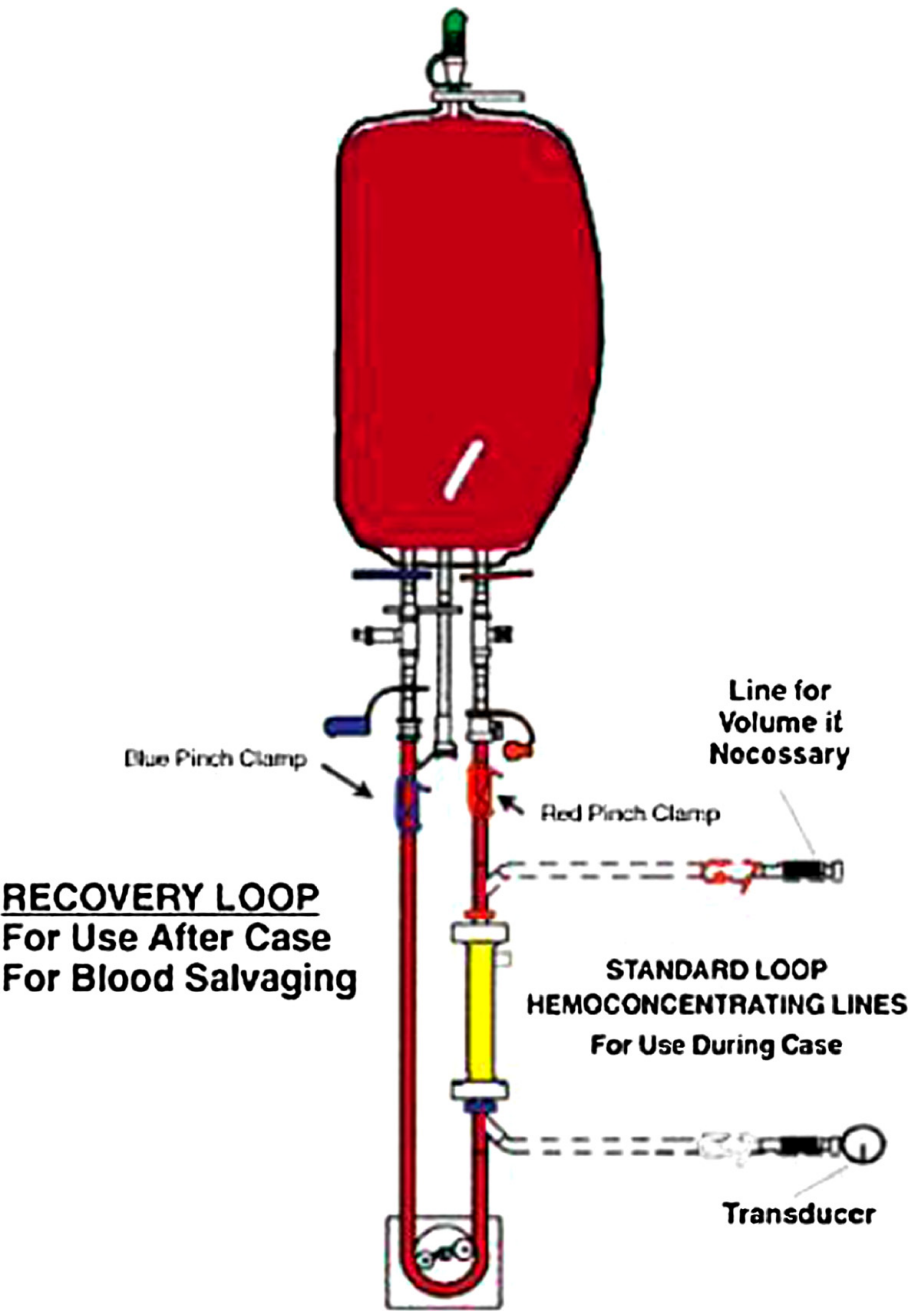
**Diagram of the “Recovery loop”, a sterile and isolated circuit (Hemobag, TS3 tubing set, pressure manometer and hemoconcentrator) to ultrafiltrate the post Cardiopulmonary bypass whole blood.** The system use a multi-pass ultrafiltration technique to concentrate blood in the Hemobag mediated by a roller pump.

The Hemobag reservoir is filled with the residual blood of the CPB reservoir and connected to the “Recovery loop”. The recovery loop consist in the connection of the Hemobag, the TS3 tubing set, the pressure manometer, the hemoconcentrator, and a roller pump that is not in use after discontinuing CPB (Figure 2). The “recovery loop” creates a sterile, and isolated circuit concentrating the whole blood into the Hemobag. Average flows are maintained around 450 mL/min, with a maximum transducer pressure of 325 mmHg. No suction is required to facilitate effluent removal.

### Statistical analysis

The Hemobag and the CW groups were compared in terms of clinical characteristic and surgical outcomes by using the Chi-square test (or the Fisher exact test when appropriate) for categorical variables and the t test for continuous variables. The significance level was set at 0.05.

In order to compare the Hemobag and the CW systems, the differences in hematocrit, platelet count, total protein concentration, albumin and fibrinogen values between pre and post treatment were calculated, both in the Hemobag and the CW groups.

## Results and discussion

Mean age was 65.7 ± 12.3 years with 40% of females (n = 25). Patients in both groups presented similar baseline clinical characteristics (Table 1). Thirty patients (60%) underwent heart valve surgery, fifteen patients (30%) underwent coronary artery bypass graft and ten patients (10%) underwent combined heart valve surgery and coronary artery bypass graft. The distribution of surgeries was equivalent in both groups.

**Table 1:** Comparison of clinical characteristics of both groups

	**Group Hemobag (n = 25)**	**Group Cell Washer (n = 25)**	**p**
Mean age (years)	65.5 ± 12.7	64.9 ± 12.1	0.865
Female	10 (40%)	10(40%)	1.000
Diabetes mellitus	9 (36%)	8 (32%)	0.765
Dislipemia	13 (52%)	15 (60%)	0.569
Hypertension	18 (72%)	17 (68%)	0.758
Peripheral vascular disease	3 (12%)	4 (16%)	1.000
Respiratory disease	4 (16%)	3 (12%)	1.000
Stroke	1 (4%)	0	1.000
NYHA III–IV class	16 (64%)	17 (68%)	0.765
Atrial fibrillation	5 (20%)	7 (28%)	0.508
Severe pulmonary hypertension (>59 mmHg)	2 (8%)	2 (8%)	1.000
Ejection fraction	57.8 ± 22	57.9 ± 32	0.990
Logistic EuroSCORE (%)	6.9 ± 8	7.5 ± 8.3	0.796

NYHA, New York Heart Association.

Surgical mortality was 4% (*n* = 2). One patient died of heart failure and one died of a stroke. There was no death during 30-day follow-up. There was no bleeding re-exploration.

The rates of major and minor postoperative morbidity were comparable among the groups. The use of vasopressor support in the ICU, were also similar among groups. Mean quantity of blood recovered and reinfused to the patient with the Hemobag device was 461 ± 174 ml and 410 ± 132 ml with the CW device (p = 0.249).

The number of blood bags transfused in the operating theater, the ICU and in the ward were similar in both groups (Table 2).

**Table 2:** Surgical outcomes of both groups

	**Group Hemobag (n = 25)**	**Group Cell Washer (n = 25)**	**p**
Cardiopulmonary bypass time (min)	89.5 ± 46.1	93.1 ± 45.3	0.782
Cross-clamp time (min)	63.3 ± 36.1	65.7 ± 39.4	0.823
Hours of intubation	2.6 ± 1.9	2.3 ± 2.0	0.589
24 h postoperative bleeding (ml)	462 ± 336	538 ± 430	0.490
Length of stay in ICU (days)	1.5 ± 2.9	1.5 ± 1.9	1.000
Total length of stay (days)	11.1 ± 10.1	11.6 ± 8.8	0.853
Hemoglobin at discharge (g/dl)	11.5 ± 1.2	10.7 ± 2.0	0.093
Death	1 (4%)	1 (4%)	1.000
Mean quantity of blood reinfused (ml)	461 ± 174	410 ± 132	0.249
Patients receiving blood transfusion	9 (36%)	11 (44%)	0.564
Patients receiving platelet transfusion	3 (12%)	5 (20%)	0.702

We have observed a global improvement of all the blood parameters analyzed in the transfusion bags before and after processing in the Hemobag group respect to the CW group (Table 3). In particular we have observed that the hematocrit increased from 21% ± 2% to 51% ± 3% in the Group H respect to the increase from 21% ± 3% to 35% ± 3% in Group CW (p < 0.001). The platelet count increase in Group H from 105 ± 12 K/mm3 to 201 ± 22 K/mm3 compared to the decrease from 102 ± 11 K/mm3 to 57 ± 11 K/mm3 in Group CW (p < 0.001). The total protein concentration passed from 3 ± 0.5 mg/dL to 9 ± 1 mg/dl in Group H and dropped from 3 ± 0.3 mg/dL to 0.4 ± 0.2 mg/dL in Group CW (p < 0.001). Albumin increased from 1.5 ± 0.3 mg/dL to 4.5 ± 0.3 mg/dL in Group H and dropped from the same 1.7 ± 0.3 mg/dL to 0.3 ± 0.1 mg/dL in Group CW (p < 0.001). Fibrinogen also increased in Group H from 125 ± 18 mg/dL to 342 ± 39 mg/dL and decreased from 122 ± 18 mg/dL to 33 ± 4 mgdL in Group CW (p < 0.001).

**Table 3:** Blood data pre and post hemoconcentration in the transfusion bags

**Variable**	**Group Hemobag**				**Group Cell Washer**				
	***MeanPre*(*SD*)**	***MeanPost*(*SD*)**	***Delta pre*-*post*(*SD*)**	***P*(*pre*-*post*) **	***Mean Pre*(*SD*)**	***Mean Post*(*SD*)**	***Delta pre*-*post*(*SD*)**	***P*(*pre*-*post*) **	***P*(*H*-*CW)***
**Hematocrit (%)**	21.52 (2.93)	51.4 (3.06)	29.88 (4.90)	<0.001	21 (2.99)	34.88 (1.96)	13.88 (3.64)	<0.001	<0.001
**Platelets (n/mm3)**	105.16 (12.92)	201.36 (22.28)	96.2 (29.04)	<0.001	102.96 (11.18)	57.64 (11.73)	−45.32 (17.23)	<0.001	<0.001
**Proteins (g/100 ml)**	3.24 (0.40)	9.48 (1.01)	6.24 (1.11)	<0.001	3.04 (0.36	0.48 (0.25)	−2.56 (0.44)	<0.001	<0.001
**Albumin (g/100 ml)**	1.55 (0.30)	4.47 (0.30)	2.92 (0.48)	<0.001	1.74 (0.35)	0.32 (0.16)	−1.42 (0.42)	<0.001	<0.001
**Fibrinogen (mg/100 ml)**	125.64 (18.20)	342.8 (39.83)	217.16 (50.62)	<0.001	122.44 (18.12)	33.88 (3.96)	−88.56 (18.61)	<0.001	<0.001

As stated in recent published STS blood conservation guidelines 2011 [9], general consensus suggests that some form of pump salvage and reinfusion of residual pump blood at the end of CPB is reasonable as part of a blood management program to minimize blood transfusion (Level of evidence C).

Processing of the circuit blood instead of direct infusion of residual pump blood is reasonable for minimizing post-CPB allogenic RBC transfusion (Level of evidence A).

As showed in the STS guidelines there are several studies in the literature that have compared the effects of direct infusion of unprocessed post-CPB blood versus centrifugation [10-17]. Despite limitations of the current body of literature (small sample size, only CABG patients), all studies have showed superiority of the pump salvage strategy compared with no salvage of residual blood. In the literature there are only few reports comparing the effectiveness of centrifugation with ultrafiltration in concentrating post CPB blood to minimize blood loss and transfusion requirements.

Boldt et al. [10] studied 40 nonrandomized patients undergoing elective CABG procedures and discovered no significant difference in blood loss or frequency of donor blood transfusion between the centrifugation and ultrafiltration groups.

Samolyk and coworkers [13] used a case-matched control study to compare centrifugation and ultrafiltration in 100 patients; there was no difference in blood utilization or postoperative bleeding between groups.

The results of the present study are in accordance with those published previously. Similarly to Samolyk et al. [13] we have analyzed the clinical and laboratory parameters of the blood bags processed with the Hemobag and conventional CW. We have not observed differences in the clinical outcomes of both groups but we have observed important differences in the quality of concentrated blood reinfused to the patient. Ultrafiltration (Group H) of residual CPB blood produced a protein-rich concentrated whole blood respect to Centrifugation (Group CW) of residual CPB blood that produced concentrated red cells mostly devoid of plasma proteins.

Experimental studies by Delaney et al and Roeder et al. [18,19] already showed that the Hemobag technique yielded significantly higher hemoglobin, hematocrit, fibrinogen, albumin, and total protein levels in the final hemoconcentrated product respect to the most common of standard method of hemoconcentration.

This report describes our initial evaluation and application of the Hemobag system confirming that its use offered an high quality hemoconcentrated blood. In our Institution the multi-disciplinary and multi-modality perioperative approach to blood conservation has become a central aspect of our general strategy to improve of our clinical results.

Part of this multi-modality approach is the hemoconcentration of the residual whole blood in the ECC post-CPB. This avoids the discard of plasma proteins, coagulation factors, and platelets as typically seen with cell washing techniques.

The introduction of the Hemobag system in our daily practice has improved the quality of blood management of our surgical procedures.

The Hemobag demonstrated to maximize the composition of the residual CPB volume to achieve a good post-CPB hemoglobin, plasma protein and coagulation factors profile for the patient. It also achieve the goal of salvaging and returning to the patient the autologous whole blood volume offering all the benefits of an appropriate colloid pressure at both macro and micro circulatory domains in terms of oxygen delivery to cells and effective homeostasis.

The Hemobag like the CW works separately from the CPB and allow surgery to continue uninterrupted while the hemoconcentration process is ongoing and to administer protamine. The CPB is maintained sterile, primed and ready to resume CPB in case of emergency. An important limitation observed for the Hemobag system respect to the CW is that it needs more time (3–5 minutes) to hemoconcentrate the same amount of residual blood post-CPB.

In our observational study the group of patients treated with the Hemobag did not required higher vasopressor support after weaning the CPB or in the postoperative time, in contrast to the reported data in the literature of patients treated with modified ultrafiltration [20]. This may have occurred because the Hemobag ultrafiltration system could be started when the patients was hemodynamic stable after weaning from CPB.

The major limitation of the present preliminary institutional report is related to the non-randomized design of the study and the small number of patients evaluated. There was no statistically significant difference in clinical outcomes in the 2 groups because the trial was not powered for this. We consider that in the future larger randomized controlled trials would help to better quantify the overall postoperative benefits to surgical patients and to better target the use of the Hemobag system for patient stability and bleeding.

## Conclusions

In summary, this preliminary institutional report demonstrated that the Hemobag system is a safe and efficient method to hemoconcentrate the residual diluted blood of the CPB compared to the CW.

## Competing interests

The authors declare that they have no competing interests.

## Authors’ contributions

AC have made conception, design, acquisition of data, interpretation of data and written the manuscript. SB performed the statistical analysis has been involved in the drafting of the manuscript revising it critically for important intellectual content. XR coordinated the study and revised critically the manuscript. All authors read and approved the final manuscript.
